# Low Physical Activity and Cardiorespiratory Fitness in People With Schizophrenia: A Comparison With Matched Healthy Controls and Associations With Mental and Physical Health

**DOI:** 10.3389/fpsyt.2019.00087

**Published:** 2019-02-28

**Authors:** Thomas W. Scheewe, Frederike Jörg, Tim Takken, Jeroen Deenik, Davy Vancampfort, Frank J. G. Backx, Wiepke Cahn

**Affiliations:** ^1^Department of Psychiatry, Rudolf Magnus Institute for Neuroscience, University Medical Center Utrecht, Utrecht, Netherlands; ^2^Department of Human Movement and Education, Windesheim University of Applied Sciences, Zwolle, Netherlands; ^3^Rob Giel Research Center, University Center of Psychiatry, University Medical Center Groningen, University of Groningen, Groningen, Netherlands; ^4^Research Department, GGZ Friesland (Friesland Mental Health Services), Leeuwarden, Netherlands; ^5^Child Development and Exercise Center, Wilhelmina Children's Hospital, University Medical Center Utrecht, Utrecht, Netherlands; ^6^GGz Centraal, Amersfoort, Netherlands; ^7^University Psychiatric Center KU Leuven, Leuven, Belgium; ^8^Department of Rehabilitation Sciences, KU Leuven, Leuven, Belgium; ^9^Department of Rehabilitation, Physical Therapy Science and Sports, Rudolf Magnus Institute for Neuroscience, University Medical Center Utrecht, Utrecht, Netherlands

**Keywords:** physical activity, sedentary behavior, cardiorespiratory fitness, schizophrenia, matched healthy controls

## Abstract

**Introduction:** The aim of this study was to objectively assess time spent in physical activity (PA) and sedentary behavior (SB) in patients with schizophrenia compared to healthy controls matched for age, gender and socioeconomic status. Associations between both PA and cardiorespiratory fitness (CRF) and mental and physical health parameters in patients with schizophrenia were examined.

**Materials and Methods:** Moderate and vigorous PA (MVPA), moderate PA, vigorous PA, total and active energy expenditure (TEE and AEE), number of steps, lying down and sleeping time was assessed with SenseWear Pro-2 body monitoring system for three 24-h bouts in patients with schizophrenia (*n* = 63) and matched healthy controls (*n* = 55). Severity of symptoms (Positive and Negative Syndrome Scale and Montgomery and Åsberg Depression Rating Scale), CRF (peak oxygen uptake, VO_2peak_), body mass index (BMI), and metabolic syndrome were assessed.

**Results:** Patients with schizophrenia performed less MVPA and moderate activity had lower TEE and AEE, spent more time per day lying down and sleeping, and had poorer CRF compared to healthy controls. The amount of MVPA, but especially CRF was associated with severity of negative symptoms in patients with schizophrenia. Only CRF was associated with BMI.

**Discussion:** The current data offer further evidence for interventions aiming to increase physical activity and decrease sedentary behavior. Given strong associations of CRF with both negative symptoms and BMI, treatment aimed at CRF-improvement may prove to be effective.

## Introduction

The premature mortality risk in patients with schizophrenia is two to three times higher compared to the general population leading to a 7–20 year reduction in life expectancy (1–3), mainly due to cardiovascular disease (4, 5). The increased cardio-metabolic risk is partly attributable to side effects of antipsychotic medication such as weight gain, dyslipidemia, and diabetes mellitus (4, 6).

Three recent meta-analyses show patients with schizophrenia engage in less physical activity (PA) (7), have high levels of sedentary behavior (SB) in their waking day (8), and have low cardiorespiratory fitness (CRF)-levels (9). The majority of the included studies used self-report to assess PA (7) which, due to recall errors and social desirability bias, has limited validity (10, 11). Illustrative, whereas no difference in PA was found using self-report measurement, accelerometry showed a large reduction of PA in patients with schizophrenia compared to healthy controls (12). As for SB, a meta-analysis demonstrated that patients with psychosis spend 11 h of their waking day being sedentary. Again, objective measurement of SB demonstrated significantly higher levels of SB compared to self-report measurements (8).

In patients with psychosis, a limited number of studies have suggested that high levels of SB and low levels of PA are associated with an increased cardio-metabolic risk [e.g., (13, 14)]. These studies did not take CRF into account, and most of these studies assessed SB and PA using self-report (10, 11). One study examined independent associations of objectively measured SB and PA with cardio-metabolic risk in inpatients with schizophrenia as well as in age/sex/body mass index-matched healthy controls (15), but failed to take CRF into account. As far as we know, only one study (16) did include CRF when investigating associations between SB and PA with cardio-metabolic risk factors in patients with psychosis. This study showed that SB is, independently of PA and CRF, associated with the individual risk factors waist and fasting blood glucose. Strikingly, CRF, even when controlled for SB and PA, remained significantly associated with clustered cardio-metabolic risk and the individual risk factor waist. The study by Bueno-Antequera et al. (16) is mildly hampered by some limitations. For instance, they did not include healthy controls, measured CRF with a submaximal test instead of a “gold standard” cardiopulmonary exercise testing (CPET), thus limiting the validity of its results (17), and had a small sample of size of outpatients, predominantly men. Therefore, they call for more research, as well as the use of (gold standard) objective measures.

The aim of this study, therefore, is to compare objectively assessed SB and PA, as well as CRF measured by CPET, in patients with schizophrenia with matched, physically inactive, but otherwise healthy controls using data from the “The Outcome of Psychosis and Fitness Therapy” study (TOPFIT).

The second aim was to determine whether SB, PA, and CRF were associated with mental and physical health parameters in both patients with schizophrenia and matched healthy controls.

## Materials and Methods

### Participants and Setting

This study included data of 63 patients with a schizophrenia spectrum disorder and 55 healthy controls, matched for gender, age, and socioeconomic status (expressed as the highest educational level of one of the parents). Patients were recruited at the University Medical Center Utrecht (Netherlands) (*n* = 26) and regional mental health care institutes (Altrecht; GGZ Duin- en Bollenstreek; GGZ Friesland) (*n* = 37). Healthy controls (*n* = 55) were recruited from the local population via advertisements. Participants were enrolled in the study between May 2007 and May 2010 and written informed consent was obtained after the procedures, and possible side effects were explained. This study was part of the TOPFIT project (“The Outcome of Psychosis and Fitness Therapy”) and registered in the ISRCTN register (http://www.controlled-trials.com/ISRCTN46241817). Patients had a diagnosis of schizophrenia (*n* = 45), schizoaffective (*n* = 15), or schizophreniform disorder (*n* = 3) according to the Diagnostic and Statistical Manual of Mental Disorders, fourth edition (DSM-IV). Diagnosis was confirmed by psychiatrists using the Comprehensive Assessment of Schizophrenia and History (CASH) (18). Patients were stable on antipsychotic medication, i.e., using the same dosage for at least 4 weeks prior to inclusion. They showed no evidence for significant cardiovascular, neuromuscular, endocrine or other somatic disorders that prevented safe participation in the study (19). Patients had no primary diagnosis of alcohol or substance abuse and had an IQ ≥ 70, as measured with the Wechsler Adult Intelligence Scale Short Form (WAIS-III SF) (20).

The inclusion criteria for the healthy controls were no diagnosis of psychiatric disorders according to DSM-IV lifetime, no first-degree relative with a psychotic or depressive disorder, and being physically inactive before inclusion (i.e., undertaking <1 h of moderate PA weekly; based on self-report). The study was approved by the Human Ethics Committee of the University Medical Center Utrecht and research committees of participating centers.

### Assessments

All measurements were assessed by a research assistant and a sports physician. Participants were asked to wear the SenseWear Pro-2 (BodyMedia, Inc., Pittsburgh, PA), body monitoring system during three 24-h time bouts (2 weekdays and 1 weekend day) except during water-based activities. This device objectively measures PA and estimates energy expenditure (21–23). The SenseWear was worn over the right arm triceps muscle and assesses minute-to-minute data through multiple sensors, namely a two-axis accelerometer and sensors measuring heat flux, galvanic skin and near body-temperature. Data are combined with gender, age, body weight, and height, to measures physical (in)activity and estimate energy expenditure using algorithms developed by the manufacturer (SenseWear Professional software, version 5.1.0.1289).

Several variables were calculated from the SenseWear data. PA was expressed in average metabolic equivalents (MET; in kcal/kg/h), an indicator of daily energy expenditure. The unit MET was used to estimate the amount of oxygen used by the body during SB and PA. Daily average time spend in total SB (<3 MET), moderate and vigorous PA (MVPA) (≥3MET), moderate (3–6 MET), vigorous (≥6 MET) were calculated from all minutes with a MET-value. Total energy expenditure (TEE; in kcals), active energy expenditure (AEE; in kcals: ≥3 MET), number of steps, lying down and sleeping time were also estimated. Data was accepted when the average on-body measuring time was at least 1,368 min per day (95% of a 24-h bout).

CRF, defined as the ability of the circulatory and respiratory systems to supply oxygen to skeletal muscles during sustained physical activity, was assessed with a cardiopulmonary exercise test (CPET), performed using a 20 watt per minute (W/min) step wise incremental protocol to exhaustion on a cycle ergometer (Lode Excalibur, Lode BV, Groningen, the Netherlands) (24). CRF was defined as the highest oxygen uptake during any 30-s interval during the test (VO2peak ml·kg^−1^·min^−1^) (25). Waist circumference (in cm) and anthropometric measurements (height in cm and weight in kg), using the same calibrated equipment in all participants, and metabolic syndrome (MetS), assessed according to the International Diabetes Foundation criteria (26), were obtained by the sports physician prior to the CPET.

To evaluate the severity of schizophrenia symptoms, the Positive and Negative Syndrome Scale (PANSS) total, positive, negative, and general (sub)scores were assessed (27). The Montgomery Åsberg Depression Rating Scale (MADRS) assessed co-morbid depressive symptoms (28). Detailed information on the amount and type of prescribed antipsychotic and other medication were gathered. Current antipsychotic medication prescribed was described in cumulative dosage and converted into haloperidol equivalents, conformable to a table from the Dutch National Health Service (29).

### Statistical Analyses

SPSS 25.0 was used to analyze the data (Armonk, NY: IBM Corp). All statistical tests were performed two-tailed and a *p* < 0.05 was considered significant. Data were examined for outliers. All analyses were performed with and without extreme outliers to examine their influence on results. In case of non-normal distribution logarithmic transformation was applied.

Multiple analyses of variance for non-categorical variables and χ^2^ analyses for categorical variables were used to examine differences between patients with schizophrenia and matched healthy controls in demographic and clinical variables. Univariate analyses were used to examine differences in SB, MVPA, moderate PA, vigorous PA, TEE, and AEE, number of steps, lying down and sleeping time, and CRF between patients and healthy controls. Gender, age, WAIS IQ-score, marital status, employment status, and Body Mass Index (BMI) were included in analyses as possible confounding factors. To investigate if differences exist between day of measurement (weekdays vs. weekend) within and between groups (patients vs. controls), repeated measures analysis of variance were performed comparing the average weekday vs. weekend day SB, MVPA, moderate PA, vigorous PA, TEE and AEE, number of steps, lying down, and sleeping time. Correction for multiple testing was applied according to the Bonferroni-correction procedure.

In patients, backward linear regression analysis (criterion: probability of F-to-remove ≥0.10) was used to assess whether the independent variables gender, age, PANSS positive, PANSS negative, PANSS general, employment status, and MADRS-score were associated with the level of SB, MVPA, and CRF (VO_2peak_ ml·kg^−1^·min^−1^). Similarly, we examined the association between physical health parameters (gender, age, employment status, BMI, haloperidol equivalent of antipsychotic medication prescribed, and number met criteria for the MetS) and SB, MVPA, and CRF (VO_2peak_ ml·kg^−1^·min^−1^). We repeated the latter regression analyses in healthy controls, examining the association between physical health parameters (gender, age, employment status, BMI, number of met criteria for MetS) and SB, MVPA, and CRF.

## Results

### Descriptive Statistics

Demographic and illness characteristics are shown in Table 1. Healthy controls had lower BMI (*p* = 0.01), waist circumference (*p* = 0.002), triglycerides (*p* < 0.001), and LDL-cholesterol (*p* = 0.02). Healthy controls were less likely to have MetS (*p* = 0.04), met on average less MetS criteria (*p* = 0.003), and smoked less cigarettes per day (*p* ≤ 0.001). Healthy controls were more likely married (*p* ≤ 0.001), had a higher IQ (*p* ≤ 0.001), and higher HDL-cholesterol levels (*p* < 0.001). No significant differences in demographic and illness characteristics, except higher diastolic blood pressure (*p* = 0.02) and lower HDL-cholesterol (*p* = 0.007), were found between male and female patients. There were no differences in type [$\chi_{\left(9\right)}^{2}$ = 5.68; *p* = 0.77] and dose [*F*_(1, 58)_ = 1.24; *p* = 0.27] of antipsychotic medication used between genders in patients.

Table 1Demographic and clinical characteristics for patients with schizophrenia and matched healthy controls.

**Table:** 

	**Patients**	**Controls**		
**Characteristic**	**(*n* = 63)**	**(*n* = 55)**		
	***N*** **(%)**	***N*** **(%)**	***F***	***p***
Gender (male)	46 (73)	36 (65)	0.79	0.37
CASH: Schizophrenia	45 (71)			
Schizo-affective disorder	15 (24)			
Schizophreniform disorder	3 (5)			
Marital status (single/married/divorced)	56/4/3	30/24/1	22.71	**<0.001**
Employment status (welfare/ working/ student/ unemployed/ unknown)	51/8/1/3/0	1/27/24/2/1	81.38	**<0.001**
Treatment (inpatient/ day hospital/ out-patients/ unknown)	9/20/33/1			
Parental education level^a^:			6.79	0.34
Primary school or less	3(5)	1 (2)		
Secondary school	37 (59)	24 (44)		
College or university degree	21 (33)	30 (54)		
Unknown	2 (3)	0		
MetS (yes)^b^	22 (35)	9 (16)	5.22	**0.04**

**Table:** 

	**Mean**	***SD***	**Mean**	***SD***	***F***	***p***
Age (year)	29.6	7.4	29.3	7.7	0.07	0.80
Height (cm)	177.9	9.2	178.2	10.1	0.03	0.86
Weight (kg)	83.0	19.2	76.3	14.3	4.51	**0.04**
BMI (kg/m^2^)	26.3	6.0	23.9	3.3	6.60	**0.01**
VO_2peak_ (ml/min/kg)	31.6	9.9	35.9	5.5	7.92	**<0.01**
WAIS Total IQ	87.2	15.6	108.1	13.8	58.13	**<0.001**
Nr. of MetS-criteria met^b^	2.3	1.4	1.5	1.2	9.55	**0.003**
Waist circumference (cm)	93.4	16.0	85.4	11.2	9.60	**0.002**
Systolic blood pressure (mm/hg)	125.4	12.6	122.8	12.2	1.33	0.25
Diastolic blood pressure (mm/hg)	76.2	9.1	74.6	9.1	0.99	0.32
Tryglicerides (mmol/L)	1.5	1.0	0.9	0.5	16.91	**<0.001**
HDL-cholesterol (mmol/L)	1.0	0.3	1.3	0.3	24.44	**<0.001**
LDL-cholesterol (mmol/L)	3.3	1.0	2.9	0.8	6.06	**0.02**
Smoking (cigarettes/day)	11.8	10.5	0.9	4.3	52.03	**<0.001**
Alcohol usage (glasses/week)	3.6	6.9	5.0	5.2	1.50	0.23
PANSS total score	62.6	10.7				
PANSS positive factor score	15.52	4.0				
PANSS negative factor score	17.46	5.8				
MADRS total score	15.16	8.4				
Duration of illness (years)	6.6	5.8				
Hospitalization until measurement (days)	193.7	265.3				
HEQ dose (mg/day)	8.1	5.2				

a *Socioeconomic status, expressed as highest level of education of one of both parents according to Roick et al. (30)*.

b *Assessed according to the International Diabetes Foundation criteria (26). CASH, Comprehensive Assessment of Schizophrenia and History; MetS, Metabolic Syndrome; BMI, Body Mass Index; VO2peak, maximum rate of oxygen consumption; WAIS, Wechsler Adult Intelligence Scale; HDL, High-density lipoproteins; LDL, Low-density lipoproteins; PANSS, Positive and Negative Syndrome Scale; MADRS, Montgomery and Åsberg Depression Rating Scale; HEQ, haloperidol equivalent. Significant differences at < 0.05 level are presented in bold*.

### Differences in SB, PA, and CRF

All variables, except SB, moderate PA, vigorous PA, and active energy expenditure data, complied with normality and homogeneity of variance demands. After logarithmic transformation of these variables, all data were analyzed parametrically. Average on-body percentage was below 95 percent in one patient with schizophrenia and three healthy controls. In total, 62 patients and 52 healthy controls, with an average on-body time of 98.3 (SD: 1.4) and 98.0 (SD: 1.2) percent, respectively, were thus included in further analyses. Results are presented in Table 2. Compared to physically inactive but otherwise healthy matched controls, patients showed significantly higher SB (*p* = 0.005), less MVPA (*p* = 0.005), and less moderate PA (*p* ≤ 0.001), but equal vigorous PA (*p* = 0.15). Patients with schizophrenia had significantly lower total (*p* = 0.001) and active (*p* = 0.002) energy expenditure compared to controls. Though the average daily number of steps taken was lower in patients with schizophrenia (mean: 8040; SD: 3072) than in controls (mean: 8884; SD: 2837), this difference did not reach significance (*p* = 0.16). Patients spent significantly more time lying down (*p* ≤ 0.001) and sleeping (*p* < 0.001) (expressed as minutes per day) than controls. Patients had significantly poorer CRF than healthy controls (*p* < 0.01). Controlling for gender, age, BMI, and marital status did not change results. Controlling for WAIS IQ led to non-significance for TEE only. However, controlling for employment status led to non-significant differences in SB, PA, and TEE and AEE, but not in lying down and sleeping time. Bonferroni-correction for multiple testing did not influence the conclusions.

**Table 2:** SB and PA in patients with schizophrenia and matched healthy controls, controlled for gender, age, and BMI influences.

	**Group**		**Test statistic**	
**Characteristic**	**Patients (*n* = 62)**	**Controls (*n* = 52)**	
	**Mean ± SD**	**Mean ± SD**	***F***	***p***
SB (<3 MET; min/day)	1303.6 ± 70.2	1254.1 ± 68.1	8.39	0.005^*^
MVPA (≥3 MET; min/day)^1^	136.4 ± 70.2	185.2 ± 68.6	8.39	0.005^*^
Moderate (3-6 MET; min/day)^1^^,^^a^	105.3 ± 72.1	152.1 ± 63.3	12.98	<0.001^*^
Vigorous (>6 MET; min/day)^1^^,^^a^	10.5 ± 20.3	16.1 ± 26.6	2.10	0.15
Total energy expenditure (kcal/day)^1^	2897 ± 582	3036 ± 455	10.74	0.001^*^
Active energy expenditure (kcal/day)^1^^,^^a^	718 ± 595	965 ± 421	9.62	0.002^*^
Steps (steps/day)^1^	8040 ± 3072	8884 ± 2837	1.97	0.16
Lying down (hours/day)^2^	11.4 ± 2.1	8.6 ± 1.2	65.95	<0.0001^*^
Sleeping time (hours/day)^2^	9.2 ± 1.9	6.5 ± 1.0	68.63	<0.0001^*^

1 *Higher score indicates superior physical activity*.

2 *Lower score indicates superior physical activity*.

a *EXP-values of logarithmically transformed and analyzed data are presented. Significant results are presented in bold*,

* *significant after Bonferoni correction for multiple testing*.

*SB, sedentary behavior; PA, physical activity; BMI, body mass index; MET, metabolic equivalent; MVPA, moderate to vigorous physical activity*.

### Differences in SB and PA on Weekdays vs. Weekend Days

Except for vigorous PA, patients and controls demonstrated significantly more SB, significantly less time on MVPA and moderate PA, had lower TEE and AEE, took fewer steps, and spent more time lying down and sleeping during the weekend compared to weekdays (Monday through Friday) (see Table 3). After Bonferroni-correction for multiple testing participants still took significantly fewer steps and spent more time lying down and sleeping. No significant differences between the two 24-h weekday assessments were found in either patients or controls for any of the SB or PA variables (all *p* > 0.20). Whereas, no differences in PA or energy expenditure were found between Saturdays or Sundays in healthy controls, patients had less MVPA (*p* = 0.04) and lower TEE (*p* = 0.005), and AEE (*p* = 0.009) on Saturdays compared to Sundays.

**Table 3:** Differences between day of measurement (weekdays vs. weekend) within (day) and between groups (day × group; patients vs. controls).

	**Group**							
**Characteristic**	**Patients (*****n*** **=** **62)**		**Controls (*****n*** **=** **52)**		**Test statistic**			
	**Weekday**	**Weekend**	**Weekday**	**Weekend**	**Day**		**Day** × **Group**	
	**Mean ± SD**	**Mean ± SD**	**Mean ± SD**	**Mean ± SD**	***F***	***p***	***F***	***p***
SB (<3 MET; min/day)	1296 ± 82	1318 ± 74	1249 ± 84	1263 ± 70	5.3	**0.02**	0.3	0.60
MVPA (≥3 MET; min/day)^1^	143.8 ± 82.2	121.6 ± 74.7	190.6 ± 84.0	174.6 ± 74.7	5.8	**0.02**	0.2	0.70
Moderate (3-6 MET; min/day)^1^	126.4 ± 75.7	106.5 ± 61.9	165.7 ± 71.2	152.6 ± 60.4	5.3	**0.02**	0.2	0.63
Vigorous (>6 MET; min/day)^1^	17.5 ± 16.6	15.2 ± 26.4	24.9 ± 23.5	21.9 ± 27.7	1.1	0.29	0.02	0.89
Total energy expenditure (kcal/day)^1^	2943 ± 634	2805 ± 621	3069 ± 522	2970 ± 475	6.5	**0.01**	0.2	0.67
Active energy expenditure (kcal/day)^1^	896 ± 546	745 ± 525	1055 ± 471	979 ± 432	4.8	**0.03**	0.5	0.47
Steps (steps/day)^1^	8565 ± 3522	6990 ± 3522	9104 ± 3251	8443 ± 3669	9.8	0.002^*^	1.6	0.20
Lying down (hours/day)^2^	11.1 ± 2.2	11.9 ± 3.3	8.1 ± 1.3	9.6 ± 2.3	17.0	<0.0001^*^	1.7	0.19
Sleeping time (hours/day)^2^	8.8 ± 2.1	9.8 ± 2.7	6.1 ± 0.9	7.3 ± 1.9	25.1	<0.0001^*^	0.2	0.62

1 *Higher score indicates superior physical activity*.

2 *Lower score indicates superior physical activity*.

*SB, sedentary behavior; MET, metabolic equivalent; MVPA, moderate to vigorous physical activity. Significant results are presented in bold*,

* *significant after Bonferoni correction for multiple testing*.

### Associations of SB, MVPA, and CRF With Mental and Physical Health

In patients, for mental health, a significant final model for SB emerged [*F*_(1, 59)_ = 4.46; *p* = 0.039; *R*^2^ = 0.069] in which PANSS negative score (beta = 0.263; *p* = 0.039) was significantly associated with SB. In the final model, gender, age, employment status, WAIS IQ, PANSS positive, PANSS general, and MADRS-score were not significantly associated with SB. This means that increasing severity of negative symptoms was associated with more SB. An identical but inversed model emerged for MVPA which means that increasing severity of negative symptoms was associated with fewer MVPA. For mental health, a significant model for CRF emerged also [*F*_(4, 56)_ = 17.195; *p* < 0.00000001; *R*^2^ = 0.551] in which gender (female vs. male; beta = −0.398; *p* < 0.0001), age (beta = −0.417; *p* < 0.0001), and PANSS negative score (beta = −0.502; *p* < 0.00001) MADRS score (beta = 0.198; *p* = 0.040) were significantly associated with CRF level indicating female gender, higher age, and more severe depressive and particularly negative symptoms were associated with poorer CRF.

In patients, for physical health, no significant final model for either SB nor MVPA emerged since none of the variables (gender, age, employment status, BMI, haloperidol equivalent of antipsychotic medication prescribed, and number met criteria for the MetS) were significantly associated with SB or MVPA, respectively. For physical health, a significant model for CRF did emerge [*F*_(4, 54)_ = 17.566; *p* < 0.00000001; *R*^2^ = 0.570] in which gender (female vs. male; beta = −0.255; *p* = 0.011), age (beta = −0.214; *p* = 0.032), employment status (beta = −2.04; *p* = 0.032), and BMI (beta = −0.489; *p* < 0.00001) were significantly associated with CRF level. This means female gender, higher age, being unemployed or on welfare, and higher BMI were associated with poorer CRF. When negative symptoms and BMI were combined in one regression model with CRF, both factors were equally related.

In healthy controls, a significant model emerged for MVPA [*F*_(7, 10)_ = 7.095; *p* = 0.002; *R*^2^ = 0.225] in which gender (female vs. male, beta = 0.307; *p* = 0.02) and BMI (beta = −0.31, *p* = 0.02) were significantly associated with MVPA. The same holds for SB [*F*_(7, 19)_ = 7.095; *p* = 0.002; *R*^2^ = 0.225; with gender −0.307, *p* = 0.02 and BMI 0.32, *p* = 0.02 being significantly associated] and CRF [*F*_(3, 48)_ = 18.101; *p* < 0.00000001; *R*^2^ = 0.531, with again gender −0.638, *p* < 0.0000001, employment status (beta = −0.210; *p* = 0.056), and BMI −0.54, *p* < 0.0001 being significantly associated with CRF]. Noteworthy, females tended to have more MVPA and less SB, but poorer CRF. The latter corresponds with the model in patients, in which also female gender and higher BMI were associated with poorer CRF.

## Discussion

This study examined objectively measured PA and inactivity, SB and CRF in patients with schizophrenia compared to inactive healthy controls. Patients with schizophrenia performed significantly less MVPA, moderate PA, more SB, had lower total and active energy expenditure, spent more time per day lying down and sleeping, and had poorer CRF compared to healthy controls. The amount of MVPA, but more prominently CRF level, was associated with the severity of negative symptoms in patients with schizophrenia. Only CRF, and not SB or MVPA, was associated with BMI.

This study adds to current knowledge by being one of the few to include CRF in studying the relationship between PA, SB and cardiovascular disease, and, more importantly, by being the only one to use the gold standard CPET in measuring CRF. CRF indeed appeared independently related to cardio-metabolic risk, more so than SB or PA. This has two implications; it stresses the importance of taking CRF into account when assessing patients' physical health status, and it implies that the implementation of interventions aiming to increase CRF is of utmost importance in tackling the alarming cardio-metabolic health of patients with schizophrenia (31). Two previous intervention studies showed this was feasible in patients with schizophrenia (32, 33). In the current study, we found an association between CRF and severity of negative symptoms, which is in line with previous research (34). The direction of this association is as of yet not exactly known; it may seem conspicuous to think negative symptoms lead to inactivity which in turn affects CRF levels. There is however emerging evidence that a bidirectional association may be possible as well. Two studies found evidence of a direct relationship of CRF (35) on cognition and PANSS symptomatology (36), respectively. In other areas, such as depression and bipolar disorder, the effect of physical activity on mood has been widely established, even though the mechanisms through which physical activity and brain functioning (mood, cognition and symptoms) affect each other are not completely understood yet. Nonetheless, this gives hope to the idea that interventions aiming to increase CRF may also reduce negative symptoms (37). This could, on its turn, have important functional benefits as well since negative symptoms evidently impact an individual's functional capacity in daily activities (38).

Our results are furthermore consistent with previous studies which reported lower levels of PA in patients with schizophrenia compared to healthy comparison subjects (30, 39–44). In line with earlier findings, we found patients with schizophrenia spend less time on moderate PA, but not on vigorous PA (43). In accordance with the only study that used doubly labeled water, the established criterion standard method for free-living energy expenditure assessment, we found reduced total and active energy expenditure in patients with schizophrenia (45).

Some limitations should be considered when interpreting present findings. First, SenseWear reliably assesses PA and energy expenditure in normal and overweight healthy adults (21–23, 46), yet has not been validated in patients with schizophrenia. SenseWear overestimated energy expenditure in obese subjects (46) and the current study included 15 obese patients and 2 obese healthy controls (BMI>30). Papazoglou et al. (46) used an older software version than the present study which was later shown to have an inferior accuracy (23). Second, as this is a cross-sectional study, only relationships between SB, PA, and CRF, and mental and physical health parameters could be examined, not causality. Third, we did not succeed in enrolling healthy controls fully matching the schizophrenia patient group, other than on age, socio-economic status and inactivity. In terms of cardiometabolic health, the patients were much worse off, which on the one hand stresses the seriousness of their health condition, but on the other hand impedes true comparison of the two groups. In addition, patients with schizophrenia and healthy subjects volunteered to engage in the study, which may have led to some selection bias because subjects motivated for PA and health improvement might have had greater interest in this study. Accordingly, this may have led to an overestimation of activity levels compared to the entire schizophrenia population. Also, the absence of a matched psychiatric control group is a limitation of our study. It would have been interesting to see whether activity patterns and CRF levels of patients with schizophrenia differ from patients with other psychiatric diagnoses. This might also shed light on the role of negative symptoms, which may be present in patients with other psychiatric disorders but are often more pronounced in patients with schizophrenia. Fourth, one could argue that the CPET is too strenuous for patients with schizophrenia. In our study, however, all controls and all but four patients with schizophrenia met maximal effort demand (RER peak ≥1.1), albeit that patients with schizophrenia did reach significantly lower average RER peak values than controls. This could however in part be due to poorer CRF and the fact that they are not accustomed to perform high-intensity exercise. Last, others often define SB as <1.5 MET whereas we defined it as <3 MET. This may have led to a higher estimate of SB.

In conclusion, our study shows patients with schizophrenia perform less PA, expend less total and active energy, spend more time lying down and sleeping, and have poorer CRF compared to physically inactive matched, healthy controls. Given the remarkably strong associations of CRF with both negative symptoms and BMI, improvement of CRF should be a primary treatment aim, which may affect both mental and physical health in patients with schizophrenia.

## Data Availability

The datasets generated for this study are available on request to the corresponding author.

## Author Contributions

TS, FB, TT, and WC conceived, designed, and amended the study and wrote the protocol. TS was responsible for the acquisition of the data. TS, FJ, and TT performed the statistical analyses. TS, FJ, and TT wrote the first draft of the manuscript. All authors provided critical review of the manuscript and approved the final version.

### Conflict of Interest Statement

The authors declare that the research was conducted in the absence of any commercial or financial relationships that could be construed as a potential conflict of interest.
